# Newsvendor problem under complete uncertainty: a case of innovative products

**DOI:** 10.1007/s10100-016-0458-3

**Published:** 2016-10-24

**Authors:** Helena Gaspars-Wieloch

**Affiliations:** Department of Operations Research, Poznań University of Economics and Business, Al. Niepodległości 10, 61-875 Poznan, Poland

**Keywords:** Newsvendor problem, Complete uncertainty, Scenario-based decision rule, Risk aversion, One-shot decision, Innovative products

## Abstract

The paper presents a new scenario-based decision rule for the classical version of the newsvendor problem (NP) under complete uncertainty (i.e. uncertainty with unknown probabilities). So far, NP has been analyzed under uncertainty with known probabilities or under uncertainty with partial information (probabilities known incompletely). The novel approach is designed for the sale of new, innovative products, where it is quite complicated to define probabilities or even probability-like quantities, because there are no data available for forecasting the upcoming demand via statistical analysis. The new procedure described in the contribution is based on a hybrid of Hurwicz and Bayes decision rules. It takes into account the decision maker’s attitude towards risk (measured by coefficients of optimism and pessimism) and the dispersion (asymmetry, range, frequency of extremes values) of payoffs connected with particular order quantities. It does not require any information about the probability distribution.

## Introduction

The newsvendor problem (NP), also known as the single-period problem (SPP) or the newsboy problem, consists in finding the order quantity which maximizes the expected profit (or minimizes the expected loss) in a single period probabilistic demand framework. This topic has attracted a great deal of attention and played a central role at the conceptual foundations of stochastic inventory theory. It was originally related to DMSU (decision making under stochastic uncertainty) or DMR (decision making under risk) where the demand is presented as a random variable with a known probability distribution. NP has been already analyzed on diverse assumptions and with various extensions (Choi 2012). Additionally, this problem has been also recently discussed in the context of DMPI—decision making with partial information (Guo 2011, 2013; Guo and Ma 2014), where the decision maker (DM) is able to subjectively define possibility degrees and satisfaction levels (the probability distribution is not known completely).

Nevertheless, according to Benzion et al. (2010), newsvendor theory should not assume that the DM faces a known distribution, since in real-life situations, the demand distribution is not always known. Furthermore, the authors demonstrate that knowing probabilities does not necessarily lead the subject closer (than that one who is unaware of the underlying demand distribution) to the optimal solution or to improve profits, see also (Besbes and Muharremoglu 2013). Therefore, the investigation of NP under complete uncertainty, i.e. with unknown probabilities (Knight 1921; Courtney et al. 1997; Sikora 2008; Trzaskalik 2008; Neumann and Morgenstern 1944; Walliser 2008), is much desired. Especially in the case of new (innovative) product development where it is quite complicated to define probabilities or even probability-like quantities, because there are no data available for forecasting the upcoming demand via statistical analysis, see also Millet (2009). It is worth emphasizing that the avoidance of probability in the case of newsvendor problem with innovative products is consistent with von Mises concept (von Mises 1998) who states that the probability of single events cannot be expressed in numbers.

Classical newsvendor models are usually based upon the assumption of risk neutrality (Khouja 1999; Lee and Nahmias 1993; Porteus 1990; Sikora 2008). Meanwhile, recently there is a growing body of literature that attempts to use alternative risk preferences rather than risk neutrality to describe the newsvendor decision-making behavior (Agrawal and Seshadri 2000; Guo and Ma 2014; Kamburowski 2014; Lee et al. 2014; Wang and Webster 2009; Wang et al. 2009, 2012; Wu et al. 2013b). In this contribution, we also take into account the DM’s attitude towards risk. That means that the solution recommended for a particular DM depends on two factors: the target objectively defined (profit maximization) and the DM’s nature.

The paper is organized as follows. Section 2 deals with the main features of the traditionally understood NP. Section 3 defines a new problem—the newsboy problem under complete uncertainty for innovative and small life cycle products. Section 4 presents a 2-criteria decision rule that may be used as a tool in searching an appropriate solution for the problem aforementioned. Section 5 provides illustrative examples. Section 6 analyzes the usefulness of other possible approaches in comparison with the approach presented in the contribution. Conclusions are gathered in the last part.

## Newsvendor problem

The classical newsvendor problem constitutes a production/procurement problem of a retailer who sells a product under random demand without keeping inventory. There are many situations in practice where keeping a product in inventory for future use is either impossible or impractical. This is the case for products such as newspapers and perishable food. A similar situation arises when an apparel retailer makes orders at the beginning of the season for a fashion item. Such orders are made for one season (sales time window) only, and any unsold (leftover) items are not kept in inventory to be sold next year. They are rather sold at deep discounts at the end of the season. Thus, in NP the retailer places an order for a product to his own supplier at the beginning of each period and the quantity procured is used solely to satisfy the demand during the current period. No inventory is kept from one period to the next (Burnetas et al. 2007). The demand for this product during the current period is not known in advance, but it is represented by a nonnegative random variable *D* with a known probability distribution. The cumulative distribution function of *D* is *F*, i.e. $P(D\le x)=F(x)$. The distribution may be:

- continuous (uniform, normal, lognormal, exponential), e.g. the endpoints of the interval with possible values of the demand are equal to $D_{min}=10$ and $D_{max}=25$, or
- discrete (the demand takes a finite and countable number of values), e.g. $P(D=1)=0.3$; $P(D=2)=0.4$; $P(D=3)=0.2$; $P(D=4)=0.1$.

**Table 1:** Profit matrix for the classical newsvendor problem (general case). *Source*: Gaspars-Wieloch (2015c)

$q \backslash D$	$D_{min}=q_{min}$	$D_{min}+1$	...	$D_{max}-1$	$D_{max}=q_{max}$	*p*(*q*)
$q_{min}$	$b\cdot q$	$b \cdot q$	$b \cdot q$	$b \cdot q$	$b \cdot q$	$p(q_{min})$
$q_{min}{+1}$	$b \cdot D-s (q-D)$	$b \cdot q$	$b \cdot q$	$b \cdot q$	$b \cdot q$	$p(q_{min}+1)$
...	$b \cdot D-s (q-D)$	$b \cdot D-s (q-D)$	$b \cdot q$	$b \cdot q$	$b \cdot q$	...
$q_{max}{-1}$	$b \cdot D-s (q-D)$	$b \cdot D-s (q-D)$	$b \cdot D-s (q-D)$	$b \cdot q$	$b \cdot q$	$p(q_{max}-1)$
$q_{max}$	$b \cdot D-s (q-D)$	$b \cdot D-s (q-D)$	$b \cdot D-s (q-D)$	$b \cdot D-s (q-D)$	$b \cdot q$	$p(q_{max})$

The goal of NP may consist in expected profit maximization or expected loss minimization. Here we focus on problems with discrete demand distributions and expected profit maximization, which are described for example in (Sikora 2008). We assume that $c_{1}$ is the unit production/purchase cost of the product. Symbol $c_{2}$ denotes the selling price (full retail price) of this product and $c_{3}$ stands for the discount price (price of leftover items/salvage value), where $c_{3 }< c_{1 }< c_{2}$. Symbol *q* signifies the quantity of supply (order quantity) and this is the decision variable. Values of $c_{1}$, $c_{2}$, $c_{3}$ allow one to calculate the unit profit (profit margin) from selling the product at price $c_{2}$: $b=c_{2}-c_{1,}$ and the unit loss from selling it at price $c_{3}$: $s=c_{1}-c_{3}$. Equation (1) enables one to compute profit *g*(*q*, *D*) gained by the retailer when the supply equals *q* and the demand is equal to *D*. Expected profit *p*(*q*) is given by Eq. (2), where $D_{min}=q_{min}$ and $D_{max}=q_{max}$ are the minimal and maximal quantity of demand considered by the retailer and *P*(*D*) is the probability that the demand will be equal to *D*. NP consists in determining $q^{*}$ which satisfies Eq. (3). In the case of discrete demand distributions, this problem may be solved by means of a profit matrix (Table 1), a recurrence equation (Eq. 4) or a critical ratio (Eq. 5), see Sikora (2008). In the recurrence equation the expected profit for *q* (i.e. *p*(*q*)) depends on the expected profit for $q-1$ (i.e. $p(q-1)$), the unit profit *b*, the unit loss *s* and the distribution function of *D* when the demand equals $q-1$ (i.e. $F(q-1$)). When computing *p*(*q*) for increasing values of *q* according to Eq. (4) we stop calculations when *p*(*q*) starts to decrease since that means that the maximal expected profit has just been reached. The critical ratio is formulated on the basis of the recurrence equation. In the critical ratio, first ratio $b/(s+b)$ is calculated and then one has to find such a value of *q* for which this ratio is at least equal to $F(q^{*}-1$) and at most equal to $F(q^{*})$.

1 $$\begin{aligned} g(q,\;D)= & {} \left\{ {\begin{array}{ll} b\cdot q &{}\quad \hbox { if }\;q\le D\;,\\ b\cdot D-s(q-D),&{}\quad \hbox { if }\;q>D\;. \\ \end{array}} \right. \end{aligned}$$

2 $$\begin{aligned} p(q)= & {} \sum _{D={D_{\mathrm{min}}}}^{{D_{\mathrm{max}}}} {g(q,D)\cdot P(D)} \end{aligned}$$

3 $$\begin{aligned} q^{*}= & {} \arg \mathop {\max }\limits _{q} p(q) \end{aligned}$$

4 $$\begin{aligned} p(q)= & {} \left\{ {{\begin{array}{lll} bq,&{}\quad if&{}\quad q=D_{\mathrm{min}}, \\ p(q-1)+b-(b+s)F(q-1),&{}\quad if&{}\quad q>D_{\mathrm{min}}. \\ \end{array} }} \right. \end{aligned}$$

5 $$\begin{aligned} F(q^{*}-1)\le & {} \frac{b}{b+s}\le F(q^{*}) \end{aligned}$$

Extended newsvendor models are variations of the classical newsvendor model involving different objectives, utility functions, supplier pricing policies, newsvendor pricing policies, discounting structures, states of information about demand and supply, constrained multi-products, multiple-products with substitution, random yields, multi-location models and different multiple production cycles (Agrawal and Seshadri 2000; Behret and Kahraman 2010; Choi 2012; Dogru et al. 2013; Gallego and Moon 1993; Goto 2013; Kamburowski 2014; Khouja 1999; King and Wallace 2012; Kocabıyıkoğlu and Popescu 2011; Petruzzi et al. 2009; Qin et al. 2011; Wang et al. 2009, 2015; Wu et al. 2013a, b).

## Newsvendor problem under complete uncertainty

We have already mentioned that in the case of new products, no relevant historical data are available for statistical demand analysis. And, as it was noticed in the introduction, Benzion et al. (2010) and Besbes and Muharremoglu (2013) underline the necessity to solve NP with an unknown distribution because such a problem much more suits real-life situations. Benzion et al. (2010) lead a behavior comparative analysis concerning decision makers aware of the underlying demand distribution and decision makers unaware of that distribution. Besbes and Muharremoglu (2013) as well as Godfrey and Powell (2001) propose some methods for the repeated newsvendor problem with unknown probabilities. In this paper we will investigate the non-repeated NP for which the probability distribution is not known (due to innovative products). Such a problem has not yet been discussed in the literature.

**Table 2:** Profit matrix for NP under complete uncertainty (general case). *Source*: Gaspars-Wieloch (2015c)

Scenarios$\backslash$alternatives	${\varvec{A}}_{1}(q=q_{min})$	${\varvec{A}}_{2 }(q=q_{min}+1)$	${\varvec{A}}_{j}$	${\varvec{A}}_{n-1}(q=q_{max}-1)$	${\varvec{A}}_{n}(q=q_{max})$
${\varvec{S}}_{1 }(D=D_{min})$	$a_{1,1}$	$a_{1,2}$	$a_{1,j}$	$a_{1,n-1}$	$a_{1,n}$
${\varvec{S}}_{2 }(D=D_{min}+1)$	$a_{2,1}$	$a_{2,2}$	$a_{2,j}$	$a_{2, n-1}$	$a_{2, n}$
${\varvec{S}}_{i}$	$a_{i,1}$	$a_{i,2}$	$a_{i,j}$	$a_{i, n-1}$	$a_{i, n}$
${\varvec{S}}_{m-1}(D=D_{max}-1)$	$a_{m-1,1}$	$a_{m-1,2}$	$a_{m-1,j}$	$a_{m-1, n-1}$	$a_{m-1, n}$
${\varvec{S}}_{m }(D=D_{max})$	$a_{m,1}$	$a_{m,2}$	$a_{m,j}$	$a_{m, n-1}$	$a_{m, n}$

When solving the non-repeated NP with unknown probabilities, one can refer to decision making with partial information, where the probability distribution is not known completely, but the demand may be characterized by possibility distributions (Guo and Ma 2014). Another approach consists in assuming that the decision is made under complete uncertainty (DMCU), which facilitates the decision making process, since this time it is not necessary to estimate probability-like quantities.

In the case of DMCU, the only parameter that should be declared (if we intend to take into consideration DM’s preferences) is the coefficient of optimism (or pessimism). The second benefit of applying DMCU to NP is the possibility to combine NP with scenario planning (Pomerol 2001; Heijden 1996), because within the framework of the newsboy problem the profit matrix can be computed in a very precise way (see Table 2). The result of the choice made under uncertainty with scenario planning depends on two factors: which decision will be selected and which scenario will occur. Thus, NP under complete uncertainty may be defined by means of a scenario-based decision model with *m* states of nature (scenarios, events): $S_{1}$, ..., $S_{i}$, ..., $S_{m}$, *n* possible alternatives (decisions, strategies, order quantities): $A=\{A_{1},\,{\ldots },\,A_{j},\,{\ldots },\,A_{n}\}$, and $n \times m$ profits ($a_{i,j}$—profit gained by the retailer if state $S_{i}$ occurs and alternative $A_{j}$ is selected) calculated according to formula (6). The distributions of payoffs are discrete. Symbols $a_{j,min}$ and $a_{j,max}$ denote the minimal and maximal profit connected with decision $A_{j}$ (see Sect. 4).

6 $$\begin{aligned} a_{ij} =\left\{ {\begin{array}{ll} b\cdot q\,,&{}\quad \hbox { if }\;j\le i\,,\\ b\cdot D-s(q-D)\,,&{}\quad \hbox { if }\;j>i\,. \\ \end{array}} \right. \end{aligned}$$

We assume that NP for new products may be an uncertain problem where a suitable pure (not mixed) strategy has to be found. In the case of pure strategies, the DM chooses and completely executes only one alternative. On the other side, a mixed strategy implies that the DM selects and performs a weighted combination of several accessible alternatives, see e.g. bonds portfolio construction, cultivation of different plants (Officer and Anderson 1968; Puppe and Schlag 2009; Sikora 2008).

There are many classical and extended decision rules designed for DMU (see e.g. Basili et al. 2005, 2008; Basili 2006; Basili and Chateauneuf 2011; Ellsberg 2001; Etner et al. 2012; García et al. 2012; Gaspars-Wieloch 2013, 2014a, b, c, d, e, 2015a, b, d, e, f; Ghirardato et al. 2004; Gilboa 2009; Gilboa and Schmeidler 1989; Hayashi 2008; Hildebrandt and Knoke 2011; Hurwicz 1952; Ioan and Ioan 2011; Marinacci 2002; Pereira et al. 2015; Perez et al. 2015; Piasecki 1990; Savage 1961; Tversky and Kahneman 1992; Wald 1950). The most known classical procedures are maximax (the optimist model), maximin (the pessimist model—Wald rule), minimax (the regret model, Savage rule), model of realism (Hurwicz rule) and Laplace criterion (model of the average, Bayes rule).

It is worth emphasizing that some of those rules (e.g. Gaspars-Wieloch 2013, 2014a, c, d, 2015b, c; Hayashi 2008; Hurwicz 1952; Ioan and Ioan 2011; Savage 1961; Wald 1950) find application when the DM intends to perform the selected strategy only once, see one-shot decisions. Others are recommended for people contemplating realization of the chosen variant many times (multi-shot decisions), e.g. Bayes rule. In the NP case with innovative and small life cycle products we certainly deal with one-shot decisions.

Some decision rules allow the DM to take into consideration his or her level of optimism (e.g. Ellsberg 2001; García et al. 2012; Gaspars-Wieloch 2014d, 2015b; Hurwicz 1952; Perez et al. 2015). Others do not give such opportunities (e.g. Hayashi 2008; Savage 1961; Wald 1950), which may be treated as a disadvantage in the case of solving the newsvendor problem under uncertainty, i.e. when future parameters are not deterministic and each decision maker has a different, individual nature.

Many extended rules mentioned above refer to the probability calculus, which is rather characteristic of DMR—decision making under risk, or DMU with known probabilities. Let us remind that in this paper we focus on the Knight’s definition, according to which uncertainty occurs when we do not know (i.e. we can not measure) the probabilities of particular scenarios (see complete uncertainty). Therefore, procedures presented for instance in Ellsberg (2001), Gilboa and Schmeidler (1989) or Tversky and Kahneman (1992) cannot be applied to the uncertain version of the newsvendor problem.

As a matter of fact, there is no unanimity in defining uncertainty. According to the first approach (see: theory of decision) the DM may choose the appropriate decision under certainty (DMC—each parameter of the problem is deterministic), under risk (DMR), with partial information (DMPI), under complete uncertainty (DMCU) or under total ignorance (DMTI). In the case of DMR, DMPI and DMCU, possible scenarios are predicted by experts or the DM. DMCU occurs when the probability of those events is not known or when the DM does not want to make use of the estimated probabilities. If the likelihood of particular scenarios is known and significant for the DM, then we deal with DMR (Haimann et al. 1985; Kaplan and Barish 1967; Knight 1921; Perez et al. 2015; Sikora 2008; Trzaskalik 2008). DMPI is characterized by partially known probabilities (Kmietowicz and Pearman 1984; Kofler and Zweifel 1993), which means that the DM knows only a) the order of scenarios or b) the intervals with possible probabilities for each scenario. DMTI concerns problems for which the DM is not able to define possible events.

Supporters of the second approach (see: theory of economics) state that uncertainty involves all situations with non-deterministic parameters, while risk means the possibility that some bad circumstances happen (potential of losing something), see Birge and Louveaux (2011); Dominiak (2009); Dubois and Prade (2012); Fishburn (1984); Ogryczak and Sliwinski (2009).

In this paper we treat uncertainty according to the first approach just to distinguish different degrees of probability knowledge, but in such expressions as “risk neutrality”, “risk aversion”, “attitude towards risk” we refer to the second approach.

In the next section we make an attempt to find an appropriate decision rule for the classical version of the newsvendor problem under complete uncertainty (NPCU).

## Two-criteria (H+B) rule for NPCU

When selecting a suitable procedure for NPCU (the likelihood of particular states is unknown) we must remember that the method should consider the DM’s nature in the decision making process. Furthermore, it is recommended to take into account the specific structure of the profit matrix of the classical NP. Table 3 presents profits for three possible cases ($b>s$, $b=s$, $b<s$), which can occur in real world situations. The first case may take place for instance when the discount price is almost equal to the purchase cost (i.e. we can recover almost the whole expenditure) and the selling price significantly exceeds the purchase (or production) cost. The first case constitutes a frequent and very advantageous situation for the newsvendor. The second case takes place when the difference between $c_{1}$ and $c_{3}$ equals the difference between $c_{2}$ and $c_{1}$. Such a situation is also advantageous, but to a lower extent. The third case occurs e.g. when the discount price is visibly lower than the purchase cost (i.e. we can recover only a part of the whole expenditure) and the selling price is almost equal to the purchase (or production) cost. The last situation is the least advantageous and occurs e.g. in the case of perishable food. Conclusions concerning the specific structure of the profit matrix of the classical NP are as follows:

- for *b* sufficiently bigger than *s*, the average of payoffs is the highest for $q=q_{max}$,
- for *b* sufficiently smaller than *s*, the average of payoffs is the highest for $q=q_{min}$,
- for $q=q_{min}$, particular profits $a_{i,j}$ are always the same (regardless of the state)—hence, the value of the profit is certain for decision $A_{1}$,
- almost all alternatives have more than one profit equal to $a_{j,max}$ and the number of such profits increases for *q* close to $q_{min}$—the higher *q* is, the less certain value $a_{j,max}$ is,
- for each decision, particular profits $a_{1,j}$, ..., $a_{i,j}$, ..., $a_{m,j}$ are always ordered in the form of a non-decreasing sequence, which means that the sets of payoffs achievable across the states almost do not overlap in the top right-hand corner of the matrix,
- the range between $a_{j,min}$ and $a_{j,max}$ is an increasing function *f*(*q*).

In connection with all those factors, we can conclude that decision rules formulated by Wald, Hurwicz, Savage, Bayes and Hayashi should not be applied to NPCU (due to the lack of possibility to consider DM’s nature, the lack of application to one-shot decisions, irrational solutions in the case of asymmetric distributions of payoffs or not overlapping sets of payoffs for particular scenarios, etc., compare with the justification presented in Gaspars-Wieloch (2012, 2014a, c) and Perez et al. (2015).

**Table 3:** Examples of profit matrix for NP under complete uncertainty ($\hbox {q}_{\mathrm{min}}=\hbox {D}_{\mathrm{min}}=1$, $\hbox {q}_{\mathrm{max}}=\hbox {D}_{\mathrm{max}}=4$). *Source*: Gaspars-Wieloch (2015c)

Examples	I. $b=5$, $s=1$				II. $b=3$, $s=3$				III. $b=1$, $s=5$			
Sc.$\backslash$ alt.	$A_{1}$	$A_{2}$	$A_{3}$	$A_{4}$	$A_{1}$	$A_{2}$	$A_{3}$	$A_{4}$	$A_{1}$	$A_{2}$	$A_{3}$	$A_{4}$
$S_{1}$	5	4	3	2	3	0	$-$3	$-$6	1	$-$4	$-$9	$-$14
$S_{2}$	5	10	9	8	3	6	3	0	1	2	$-$3	$-$8
$S_{3}$	5	10	15	14	3	6	9	6	1	2	3	$-$2
$S_{4}$	5	10	15	20	3	6	9	12	1	2	3	4

Therefore, we use in this paper a less-known extended procedure devoted to DMU, i.e. a hybrid of Hurwicz and Bayes rules (H+B rule), which is described in detail in Gaspars-Wieloch (2014a, 2015a). This method, thanks to parameters $\alpha \in [0,1]$ (the coefficient of pessimism is close to 1 for extreme pessimists—risk averse behaviour) and $\beta =1-\alpha \in [0,1]$ (the coefficient of optimism is close to 1 for radical optimists—risk prone behaviour), takes into account DM’s preferences. Both coefficients are not measures of probability—they just subjectively present someone’s behaviour (attitude). In the H+B rule, in contradiction to the Hurwicz, Wald, Hayashi, Savage approaches, all outcomes have an influence on the value of the final measure, which is quite advantageous for cases where alternatives contain many profits almost equal to extreme values. The final measure is a weighted average of all payoffs and weights depend on the DM’s nature. The general idea of H+B is to assign, for a pessimist, $\alpha$ to the last term of the non-increasing sequence of all payoffs related to a given decision and $\beta$ to the remaining terms of that sequence. For an optimist, weights are set in a different way: $\beta$ is connected with the first term of the sequence and $\alpha$ with the remaining ones. Due to the fact that, in the newsvendor problem, the ranges of profits related to particular alternatives vary rather significantly, we will support the H+B rule for NPCU with an additional auxiliary decision tool, which analyzes the deviations between outcomes (Gaspars-Wieloch 2015c). The use of the standard deviation as a supplementary tool in the scenario-based decision making process has been also proposed for instance by Ioan and Ioan (2011) and Piasecki et al. (2013). The second criterion (standard deviation, see Eqs. 11–12, step 7) is introduced in the final step of the procedure in order to find a relatively safe strategy (i.e. an alternative with a relatively small range of payoffs and with as little negative payoffs as possible), which is especially crucial in the case of cautious DMs. The deviation criterion is only applied to decisions with the highest index $hb_j$. However, if there are other decisions with indices very close to the highest one, we recommend to calculate and compare the value of the second measure for the whole subset containing the best strategies according to the first criterion. We purposely do not define the acceptable distance between the highest index $hb_{j}$ and the other ones. Let it be within the competence of the DM.

The suggested 2-criteria (H+B) rule for NPCU may consist of the following steps:

1. Determine $\alpha$ and $\beta$ (subjectively or on the basis of psychological tests). If $\alpha \in [0,0.5)$, then $\alpha =\alpha _o,\beta =\beta _o$ (${\alpha }_{o}$ and ${\beta }_{o}$ are optimist’s coefficients). If $\alpha \in (0.5,1]$, then $\alpha =\alpha _p,\beta =\beta _p$ (${\alpha }_{p}$ and ${\beta }_{p}$ are pessimist’s coefficients).
2. Define $q_{min}=D_{min}$, $q_{max}=D_{max}$, *m*, *n* and the set of alternatives (*A*).
3. Estimate prices $c_{1,}c_{2}$, $c_{3}$, compute *b*, *s* and generate the profit matrix.
4. Find a non-increasing sequence of gains $Sq_j =(a_{1,j},\ldots ,a_{t,j},\ldots ,a_{z,j})$ for each order: $a_{t,j}\ge a_{t+1,j}$ ($t=1,{\ldots },z-1)$, *z*—number of terms in the sequence, *t*—number of the term in the sequence.
5. Calculate, for each decision, index $hb_j(hb_j^p$, $hb_{j}^{o}$ or $hb_j^{0.5}$ depending on parameter $\alpha$). If $\alpha \in (0.5,1]$, calculate $hb_j^p$ (index for pessimists) according to Eq. (7). If $\alpha \in [0,0.5)$, compute $hb_j^o$ (index for optimists) following formula (8). If ${\alpha } =0.5$, calculate $hb_j^{05}$ using Eq. (9), where $b_{j }$denotes the Bayes criterion, i.e. the average of all payoffs.
  7 $$\begin{aligned} hb_j^p= & {} \frac{\alpha _p \cdot a_{z,j} +\beta _p \cdot \sum \nolimits _{t=1}^{z-1} {a_{t,j} } }{\alpha _p +(z-1)\cdot \beta _p } \end{aligned}$$
  8 $$\begin{aligned} hb_j^o= & {} \frac{\alpha _o \cdot \sum \nolimits _{t=2}^z {a_{t,j} } +\beta _o \cdot a_{1,j} }{(z-1)\cdot \alpha _o +\beta _o } \end{aligned}$$
  9 $$\begin{aligned} hb_j^{0.5}= & {} hb_j^p =hb_j^o =b_j =\frac{1}{m}\cdot \sum _{i=1}^m {a_{i,j}} \end{aligned}$$
  The denominators in Eqs. (7–8) are introduced so that the final value of particular indices belongs to interval [$$w_{j},M_{j}$$], where $$w_{j}$$ and $$M_{j}$$ are the values of Wald criterion and maximax criterion, respectively, i.e. the last ($$a_{z,j})$$ and the first ($$a_{1,j})$$ term of $${Sq}_{j}$$. Denominators are not crucial—they can be omitted when preparing the ranking.
6. Choose alternative $A^{*}_{j}$ fulfilling condition (8).
  10 $$\begin{aligned} A_{j}^{*} =\mathop {\arg \max }\limits _{j} (hb_j ) \end{aligned}$$
7. If set $$A^{*}$$ containing decisions $$A^{*}_{j}$$ is a singleton set, stop the procedure ($$A^{*}_{j}$$ is the appropriate solution). Otherwise, select decision $$A^{**}_{j}$$ which satisfies Eqs. (11–12).
  11 $$\begin{aligned} A_j^{**}= & {} \mathop {\arg \min }\limits _{{j^{*}}} (\sigma _{{j^{*}}} ) \end{aligned}$$
  12 $$\begin{aligned} \sigma _j= & {} \sqrt{\frac{1}{m}\sum \nolimits _{i=1}^m {\left( {a_{i,j} -\overline{a_j } } \right) ^{2}} } \end{aligned}$$
  where $\sigma _{{j^{*}}}$ is the standard deviation calculated for all decisions $A^{*}_j$ . The deviation criterion is only applied to decisions with the highest index $hb_j$. Nevertheless, as it was already mentioned, if there are other decisions with indices very close to the highest one, we recommend to compare the value of the second measure for the whole subset containing the best strategies according to the first criterion.

In the last part of Sect. 4 we would like to explain in detail terms and equations given in steps 5 and 7 of the algorithm presented above.

The assignment of parameters $\alpha$ and $\beta$ to particular payoffs, depending on the level of optimism (see Eqs. 7–9, step 5), is justified in Gaspars-Wieloch (2014a) where the author suggests a significant modification of the classical Hurwicz’s decision rule and adds to that procedure some features characteristic of Bayes rule. That is why, the approach proposed in Gaspars-Wieloch (2014a) is a one-criterion hybrid of Hurwicz and Bayes rules. In the aforementioned article as well as in Gaspars-Wieloch (2014c) we can find examples for which the original Hurwicz decision rule leads to illogical choices because it does not take into consideration the dispersion of payoffs. Let us analyze the following case in order to illustrate the drawback of Hurwicz rule. The DM should select one out of two alternatives A1 and A2 with payoffs (5,1,1,1,1,1) and (1,4.8, 4.7, 4.6, 4.5, 4.4) respectively. If the DM is a moderate optimist, let us say ${\alpha }=0.4$ and $\beta =0.6$, Hurwicz rule will recommend A1 since $H_{1}=0.4 \cdot 1+0.6\cdot 5=3.4$ and $H_{2}=0.4 \cdot 1+0.6 \cdot 4.8=3.28$. Note that this choice is quite astonishing because our DM is not a radical optimist. Hence, he or she ought to look for a safer decision, i.e. an alternative which gives relatively good results not only if the best scenario occurs, but also if other states take place. The answer suggested by Hurwicz rule results from the fact that the procedure takes into account extreme values only and not the specificity of the whole set of gains. That example was an incentive to modify the original approach so that it suggests rational decision variants for any decision problem. In Gaspars-Wieloch (2014a) the author gets to the point that if the index value should depend on all payoffs (not only on the extreme ones), parameters $\alpha$ and $\beta$ have to be assigned to all of them (not only to the extreme ones) and such a feature is typical of Bayes rule. In connection with the fact that a radical optimist rather focuses on the highest payoff, a radical pessimist rather focuses on the smallest payoff and a moderate DM mainly analyzes intermediate profits, the assignment of coefficients is supposed to be different, depending on the DM’s nature. Therefore, in the author’s opinion, in the case of optimists the high coefficient of optimism should be assigned to $a_{j,max}$ and the small coefficient of pessimism should be assigned to the remaining gains (optimists expect the occurrence of the best scenario, so it should have the biggest weight). In the case of pessimists the high $\alpha$ ought to be multiplied by $a_{j,min}$ and the small $\beta$ ought to be multiplied by the other profits. In the case of moderate DMs with parameters ${\alpha } = {\beta } = 0.5$, it does not matter which Eq. (7 or 8) will be applied, since the use of both of them boils down to equal weights for each gain. The approach described above allows one to take into consideration the frequency of extreme (or nearly extreme) values. Of course, we may ask a question, why parameters $\alpha$ and $\beta$ should be assigned to particular payoffs in the way described above. The answer is that if we assign them in an inverse way, i.e. for optimists the low $\alpha$ to $a_{j,min}$ and the high $\beta$ to the other profits, and for pessimists the low $\beta$ to $a_{j,max}$ and the high $\alpha$ to the remaining gains, we will assume that the pessimist expects, apart from the lowest one, the occurrence of quite high profits (without the highest one) and that the optimist, apart from the highest one, expects the occurrence of quite low profits (without the lowest one), which is illogical.

The idea of the hybrid presented in Gaspars-Wieloch (2014a) is to recommend for a strong pessimist an alternative with a relatively high payoff $a_{j,min}$ or with quite frequent payoffs (almost) equal to $a_{j,max}$. On the other hand, that rule suggests for an strong optimist an alternative with the highest (or almost the highest) payoff $a_{j,max}$, but its highest payoffs do not have to be frequent.

It is worth emphasizing that in the procedure proposed in Gaspars-Wieloch (2014a) the index value depends on the number of states of nature, which is not the case of Hurwicz rule. For pessimists, when the set of scenarios increases, the importance of payoff $a_{j,min}$ in the index decreases and the significance of the remaining profits increases. On the other hand, for optimists, the importance of payoff $a_{j,max}$ decreases and the significance of the remaining profits increases. Hence, again, we can observe the impact of Bayes rule in the analyzed hybrid, because the chance of the occurrence of a given event decreases along with the growth of the number of states of nature.

One of the advantages of the hybrid described in Gaspars-Wieloch (2014a) is the fact that for extreme optimists (${\beta }=1$) and extreme pessimists (${\alpha }=1$) solutions recommended by that procedure are the same as those suggested by Hurwicz rule.

In this contribution, we use the term “appropriate solution” or “suitable solution” (step 7) to describe an alternative which maximizes the $hb_j$ index value and which, in case of the occurrence of multiple solutions, minimizes the standard deviation calculated merely for decisions with the highest index. Thus, note that the adjective “appropriate” is not related to the real profit gained by the newsvendor, but it is connected with the DM’s nature. “Appropriate” means “which suits to his or her degree of optimism/pessimism”.

It is assumed in this article that an appropriate solution for radical pessimists is an alternative for which:

- the smallest gain is bigger, equal or almost as big as the smallest gains of the remaining decisions, and/or
- the subset of gains almost equal to $a_{j,max }$is numerous,

since the pessimist fears the worst, regardless of the decision selected, and that is why, such a DM needs an alternative which is attractive even if the worst state occurs and which gives a feeling of security.

We also assume that a suitable solution for radical optimists is an alternative for which:

- the highest gain is bigger, equal or almost as big as the highest gains of the remaining decisions, and
- the subset of gains almost equal to $a_{j,max }$does not need to be numerous,

since the optimist is almost or even completely sure that, regardless of the decision selected, the best scenario will occur.

To sum up, the more the DM is an optimist, the more the rule favours alternatives with the highest payoff $a_{j,max}$ (no matter what the remaining gains connected with those decisions are). On the other hand, the more the DM is a pessimist, the more the rule favours variants with the highest payoff $a_{j,min}$ (no matter what the remaining gains connected with those decisions are).

## Illustrative examples

In this section we are going to solve several problems (Examples 1–8) by means of the 2-criteria (H+B) rule. The first four examples are based on data presented in Table 3 (first column), but each example analyzes a different type of DM. The next four examples also refer to the same initial data (Table 3, first column), but this time the number of scenarios is not equal to the number of alternatives.

### Example 1

First, we assume that the DM is almost an extreme pessimist:

1. $${\alpha }=0.9$$, $${\beta }=0.1$$. Thus $$\alpha =\alpha _p,\beta =\beta _p$$.
2. $$q_{min}=D_{min}=1$$, $$q_{max}=D_{max}=4$$, $$m=n=4$$, $$A=\{A_{1}, A_{2}, A_{3}, A_{4}\}$$.
3. $c_{1}=6_{,} c_{2}=11$, $c_{3}=5$. Hence $b=5$, $s=1$. The profit matrix is given in Table 3 (first column).
4. $$Sq_{1}=(5,5,5,5)$$, $${Sq}_{2}=(10,10,10,4)$$, $${Sq}_{3}=(15,15,9,3)$$, $${Sq}_{4}=(20,14,8,2)$$.
5. Indices $hb_j^p$ are calculated in the following way:
  $$\begin{aligned} hb_1^p= & {} \frac{0.9\cdot 5+0.1\cdot \left( {5+5+5} \right) }{0.9+3\cdot 0.1}=5.0;\\ hb_2^p= & {} \frac{0.9\cdot 4+0.1\cdot \left( {10+10+10} \right) }{0.9+3\cdot 0.1}=5.5;\quad hb_3^p =5.5;\quad hb_4^p =5.0 \end{aligned}$$
6. There are two alternatives $A^{*}_{j}$: $A^{*}=\{A_{2},A_{3}\}$ (see Eq. 10).
7. Set $$A^{*}$$ is not a singleton set. Thus, it is necessary to compute $${\sigma }_{{j^{*}}}$$: $${\sigma }_{2}=3.0$$, $${\sigma }_{3}=5.74$$. Decision $$A_{2}$$ is the appropriate alternative ($$A^{**}_{j})$$ since it has the highest index $$hb_j^p$$ (in set *A*) and the lowest standard deviation (in set $$A^{*})$$. The order quantity should be equal to 2.

### Example 2

Now, let us analyze the same case (Table 3, first column) for a moderate decision maker: ${\alpha }=0.5$, ${\beta }=0.5$. Indices $hb_j$ are calculated as follows:

$$\begin{aligned} hb_1= & {} \frac{5+5+5+5}{4}=5.0;\quad hb_2 =\frac{4+10+10+10}{4}\\= & {} 8.5;hb_3 =10.5;hb_4 =11 \end{aligned}$$

There is one alternative $$A^{*}_{j}$$: $$A^{*}=\{A_{4}\}$$. Assume that the DM does not treat value 10.5 ($${hb}_{3})$$ as very close to 11 ($${hb}_{4})$$. Hence, decision $$A_{4}$$ is the appropriate alternative. The order quantity should be equal to 4.

### Example 3

If the problem is still described by Table 3 (first column) and the decision maker is a moderate pessimist with parameters, e.g. ${\alpha }=0.62$, ${\beta }=0.38$, indices are equal to $hb_1^p =5$, $hb_2^p =8.339$, $hb_3^p =10,018$, $hb_4^p =10,036.$ As a matter of fact, set $A^{*}=\{A_{4}\}$ is a singleton. Nevertheless, index $hb_p$ for alternative $A_{3}$ is almost equal to the highest value. Therefore, the DM may want to take into consideration that decision in the last step of the procedure. We compute the standard deviation: ${\sigma }_{3}=5.74$, ${\sigma }_{3}=7.75$. The order quantity should be equal to 3.

### Example 4

If the DM is almost a radical optimist, e.g. ${\alpha }=0.1$ (and profits are equal to values presented in Table 3, first column), then indices $hb_j^o$ are equal to 5, 9.5, 13.5 and 17, respectively. In that case, decision $A_{4}$ ought to be the best one.

In the first part of this section, we have considered only situations where $q_{min}=D_{min}$, $q_{max}=D_{max}$, since step 2 of the decision rule is based on such an assumption. However, it is quite interesting to analyze other cases for which: $q_{min}<D_{min}$, $q_{min}>D_{min}$, $q_{max}<D_{max}$, $q_{max}>D_{max}$, see Examples 5–8.

### Example 5

Example 5 (Table 4) concerns the case where $q_{min}<D_{min}$, $q_{max}=D_{max}.$ Parameters $q_{min}=1$, $D_{min}=2$, $q_{max}=D_{max}=4$ signify that the DM treats three demand quantities as possible (2,3,4), but he or she is willing to order less than the lowest demand ($1<2$). Note that in such circumstances decision $A_{1}(q=q_{min})$ will be always dominated by $A_{2}(q=q_{min}+1)$, since $a_{1,2}>a_{1,1}$, $a_{2,2}>a_{2,1}$ etc. Thus, there is no need to add in the analysis an order quantity lower than the lowest possible demand quantity, since neither 2-criteria (H+B) rule nor other rules will recommend that order quantity.

**Table 4:** Example 5: $q_{min}<D_{min}$ ($\hbox {q}_{\mathrm{min}}=1$, $\hbox {D}_{\mathrm{min}}=2$, $\hbox {q}_{\mathrm{max}}=\hbox {D}_{\mathrm{max}}=4$). *Source*: Prepared by the author

Example 5	$b=5$, $s=1$			
Sc.$\backslash$alt.	$A_{1}$	$A_{2}$	$A_{3}$	$A_{4}$
$S_{1}$	5	10	9	8
$S_{2}$	5	10	15	14
$S_{3}$	5	10	15	20

### Example 6

Example 6 (Table 5) is related to the case $q_{min}>D_{min}$, $q_{max}=D_{max}.$ Parameters $q_{min}=2$, $D_{min}=1$, $q_{max}=D_{max}=4$ mean that the decision maker does not intend to consider the order quantity equal to the lowest possible demand quantity, which entails a natural elimination of the best strategy for a radical pessimist. Nevertheless, especially for newsvendors-optimists, such an approach is justifiable.

**Table 5:** Example 6: $q_{min}>D_{min}$ ($\hbox {q}_{\mathrm{min}}=2$, $\hbox {D}_{\mathrm{min}}=1$, $\hbox {q}_{\mathrm{max}}=\hbox {D}_{\mathrm{max}}=4$). *Source*: Prepared by the author

Example 6	$b=5$, $s=1$		
Sc.$\backslash$alt.	$A_{1}$	$A_{2}$	$A_{3}$
$S_{1}$	4	3	2
$S_{2}$	10	9	8
$S_{3}$	10	15	14
$S_{4}$	10	15	20

### Example 7

Example 7 (Table 6) is devoted to the case: $q_{max}<D_{max}$, $q_{min}=D_{min}.$ Parameters $q_{min}=1$, $D_{min}=1$, $q_{max}=3$, $D_{max}=4$ signify that the decision maker does not want to take into account the order quantity equal to the highest possible demand quantity, which, this time, entails a natural elimination of the best strategy for an extreme optimist. Hence, especially for newsvendors-pessimists, such an approach is also justifiable.

**Table 6:** Example 7: $q_{max}<D_{max}$ ($\hbox {q}_{\mathrm{min}}=1$, $\hbox {D}_{\mathrm{min}}=1$, $\hbox {q}_{\mathrm{max}}=3$, $\hbox {D}_{\mathrm{max}}=4$). *Source*: Prepared by the author

Example 7	$b=5$, $s=1$		
Sc.$\backslash$alt.	$A_{1}$	$A_{2}$	$A_{3}$
$S_{1 }$	5	4	3
$S_{2 }$	5	10	9
$S_{3}$	5	10	15
$S_{4 }$	5	10	15

### Example 8

Finally, Example 8 (Table 7) is connected with the case: $q_{max}>D_{max}$, $q_{min}=D_{min}.$ Parameters $q_{min}=1$, $D_{min}=1$, $q_{max}=4$, $D_{max}=3$ mean that the DM treats three demand quantities as possible (1,2,3), but he or she is willing to order more than the highest demand ($4>3$). Note that for high values of the coefficient of optimism, the 2-criteria (H+B) decision rule will suggest the highest order quantity $q_{max}$. However, from a logical point of view, the choice of an order quantity exceeding the highest possible demand quantity is quite unreasonable. Payoffs connected with $q>D_{max}$ are smaller than payoffs related to $q=D_{max}.$

**Table 7:** Example 8: $q_{max}>D_{max}$ ($\hbox {q}_{\mathrm{min}}=1$, $\hbox {D}_{\mathrm{min}}=1$, $\hbox {q}_{\mathrm{max}}=4$, $\hbox {D}_{\mathrm{max}}=3$). *Source*: Prepared by the author

Example 8	$b=5$, $s=1$			
Sc.$\backslash$alt.	$A_{1}$	$A_{2}$	$A_{3}$	$A_{4}$
$S_{1}$	5	4	3	2
$S_{2}$	5	10	9	8
$S_{3}$	5	10	15	14

Other examples, not presented in this contribution, could be related to the following general case: $q_{min}\ne D_{min}$ and $q_{max}\ne D_{max}$.

The analysis of all cases for which the maximal or minimal order quantity does not overlap with the maximal or minimal demand quantity, respectively, allows us to conclude that decision problems with $q_{min}>D_{min}$ or $q_{max}<D_{max}$ may be also considered. On the other hand, decision problems for which $q_{min}<D_{min}$ or $q_{max}>D_{max}$ should not be analyzed.

## Other possible approaches for NPCU: comparative analysis

As we could notice in previous sections, the non-repeated newsvendor problem with unknown probabilities is worth investigating in the case of innovative products. We also stated that NP under uncertainty has been already discussed in the literature, but not on the assumption that the decision is made just for one period and that the DM’s coefficient of optimism is taken into account in the decision making process. We suggested the use of a hybrid of Bayes and Hurwicz rules supported by a supplementary standard deviation criterion due to a very specific dispersion of payoffs. However, the approach presented in this paper is just a suggestion.

In this section we will have the opportunity to compare the results obtained by means of diverse decision rules designed for one-shot decisions and enabling one to use the coefficients of optimism and pessimism, i.e. Hurwicz method, SAPO decision rule (Gaspars-Wieloch 2014c) and SF+AS method (Gaspars-Wieloch 2015b). The Hurwicz rule takes into account only extreme values. SAPO procedure considers averages of the first several and the last several terms of non-increasing sequences of payoffs—the cardinality of those subsets depend on DM’s preferences. Those averages are multiplied by the coefficient of optimism and the coefficient of pessimism, respectively, and some weights dependent on the cardinality of aforementioned subsets. SF+AS method reduces the initial set of states of nature in order to make the decision on the basis of those scenarios which correspond to the DM’s nature. In that procedure the status (pessimistic, moderate or optimistic) of a given state of nature does not vary depending on the alternative, but is fixed for all decisions. It is assumed that the higher the sum of the so-called “dominance cases” for a given scenario is, the more optimistic this scenario should be. After selecting a decision on the basis of a reduced set of events, the chosen alternative is additionally checked in terms of the number of its payoffs bigger than the maximal Wald criterion. The more pessimist the DM is, the more numerous that number should be. Thus, SF+AS provides a kind of security for pessimists and moderate DMs.

Table 8 presents rankings of decisions for the first four examples analyzed in the contribution (Table 3, first column, $b=5$, $s=1$). Rankings are generated on the basis of four procedures. In the case of SAPO method, it is additionally required to set arbitrarily parameters $d_{j}^{min}$ and $d_{j}^{max}$, which denote the allowable degree of deviation from $a_{j,min}$ and $a_{j,max}$, respectively. The cardinality of subsets of the lowest results and the highest results is determined by two factors (degrees of deviation and coefficients of optimism/pessimism). In the case of SF+AS, calculations are preceded by the assignment of a suitable interval for $\beta$ to each scenario (Table 9). The last column of Table 8 indicates states of nature on the basis of which the final decision is made.

Results in Table 8 allow us to draw the following conclusions:

1. For Hurwicz and SAPO methods, rankings (but not values) are the same, which is due to the fact that both methods focus on extreme values (Hurwicz) or subsets of extreme values (SAPO), and which is also due to a small number of states of nature in the analyzed problem. More significant differences can be observed when the set of scenarios increases.
2. 2crit(H+B) is the only rule which does not recommend $A_{1}$ as the best choice in the case of ${\alpha }=0.9$. This rule suggests $A_{1}$, but for $\alpha \ge 0.94$, i.e. for more radical pessimists.
3. The most visible discrepancies occur between SF+AS and the remaining rules, which is caused by totally different initial assumptions. The last column of Table 8 clearly shows for instance that in Example 1 scenario $\hbox {S}_{1}$ is the only one having an impact on the final choice. Other states of nature are not taken into account.

**Table 8:** Results obtained for different methods (b = 5, s = 1). *Source*: Prepared by the author

Examples$\backslash$methods	2-crit(H+B)	Hurwicz	SAPO ${(\hbox {d}}^{\mathrm{min}}=0.4$ ${\hbox {d}}^{\mathrm{max}}=0.4)$	SF+AS	
Ex I ($\upalpha = 0.9$)	$\hbox {A}_{2}$ (5.5 / 3.0)	$\hbox {A}_{1}$ (5.0)	$\hbox {A}_{1}$ (5.0)	$\hbox {A}_{1}$ (5.0)	$\hbox {S}_{1}$
	$\hbox {A}_{3}$ (5.5 / 5.74)	$\hbox {A}_{2}$ (4.6)	$\hbox {A}_{2}$ (4.6)	$\hbox {A}_{2}$ (4.0)	
	$\hbox {A}_{1}$ (5.0)	$\hbox {A}_{3}$ (4.2)	$\hbox {A}_{3}$ (4.2)	$\hbox {A}_{3}$ (3.0)	
	$\hbox {A}_{4}$ (5.0)	$\hbox {A}_{4}$ (3.8)	$\hbox {A}_{4}$ (3.8)	$\hbox {A}_{4}$ (2.0)	
Ex II ($\upalpha$ = 0.5)	$\hbox {A}_{4}$ (11.0)	$\hbox {A}_{4}$ (11.0)	$\hbox {A}_{4}$ (12.5)	$\hbox {A}_{2}$ (10.0)	$\hbox {S}_{2}$
	$\hbox {A}_{3}$ (10.5)	$\hbox {A}_{3}$ (9.0)	$\hbox {A}_{3}$ (10.88)	$\hbox {A}_{3}$ (9.0)	
	$\hbox {A}_{2}$ (8.5)	$\hbox {A}_{2}$ (7.0)	$\hbox {A}_{2}$ (9.5)	$\hbox {A}_{4}$ (8.0)	
	$\hbox {A}_{1}$ (5.0)	$\hbox {A}_{1}$ (5.0)	$\hbox {A}_{1}$ (5.0)	$\hbox {A}_{1}$ (5.0)	
Ex III ($\upalpha$ = 0.62)	$\hbox {A}_{3}$ (10.02 / 5.74)	$\hbox {A}_{4}$ (8.84)	$\hbox {A}_{4}$ (10.40)	$\hbox {A}_{2}$ (8.98)	$\hbox {S}_{2}$, $\hbox {S}_{1}$
	$\hbox {A}_{4}$ (10.04 / 7.75)	$\hbox {A}_{3}$ (7.56)	$\hbox {A}_{3}$ (8.99)	$\hbox {A}_{3}$ (7.98)	
	$\hbox {A}_{2}$ (8.34)	$\hbox {A}_{2}$ (6.28)	$\hbox {A}_{2}$ (8.18)	$\hbox {A}_{4}$ (6.98)	
	$\hbox {A}_{1}$ (5.00)	$\hbox {A}_{1}$ (5.00)	$\hbox {A}_{1}$ (4.70)	$\hbox {A}_{1}$ (5.0)	
Ex IV ($\upalpha$ = 0.1)	$\hbox {A}_{4}$ (17.0)	$\hbox {A}_{4}$ (18.2)	$\hbox {A}_{4}$ (18.2)	$\hbox {A}_{4}$ (20.0)	$\hbox {S}_{4}$
	$\hbox {A}_{3}$ (13.5)	$\hbox {A}_{3}$ (13.8)	$\hbox {A}_{3}$ (13.8)	$\hbox {A}_{3}$ (15.0)	
	$\hbox {A}_{2}$ (9.5)	$\hbox {A}_{2}$ (9.4)	$\hbox {A}_{2}$ (9.4)	$\hbox {A}_{2}$ (10.0)	
	$\hbox {A}_{1}$ (5.0)	$\hbox {A}_{1}$ (5.0)	$\hbox {A}_{1}$ (5.0)	$\hbox {A}_{1}$ (5.0)

**Table 9:** Payoff matrix, sums of “dominance cases” and intervals for $\beta$ (for b = 5, s = 1). *Source*: Prepared by the author

Scenarios	Alternatives				Sum of “dominance cases” ($d_{i}$)	Interval for $\beta$
	$A_{1}$	$A_{2}$	$A_{3}$	$A_{4}$		
$S_{1}$	5	4	3	2	0	[0.00, 0.14]
$S_{2}$	5	10	9	8	3	]0.43, 0.57]
$S_{3}$	5	10	15	14	5	]0.71, 0.86]
$S_{4}$	5	10	15	20	6	]0.86, 1.00]

For the sake of completeness, let us analyze Examples IX–XII for data given in Table 3 in the last column ($b=1$, $s=5$), see Tables 10 and 11. Note that in the newsvendor problem the change of parameters *b* and *s* does not affect the sum of “dominance cases” for particular events (compare Tables 9, 11).

Results in Table 10 allow us to draw the following conclusions:

1. For Hurwicz and SAPO methods, rankings (but not values) are again the same for the reasons described above.
2. 2crit(H+B) is the only rule which does not recommend $A_{4}$ as the best choice in the case of ${\alpha }=0.1$. This rule suggests $A_{4}$, but for $\alpha \le 0.06$, i.e. for more radical optimists. Such recommendations are totally justified since the order quantity equal to 4 leads to a positive payoff only if the demand also equals 4 (or more). In other cases that strategy leads to losses (even −14!), which is extremely undesired (if we extend the size of the problem, let us say: $\hbox {q}_{\mathrm{min}}=\hbox {D}_{\mathrm{min}}=1$, $\hbox {q}_{\mathrm{max}}=\hbox {D}_{\mathrm{max}}=50$, then the highest loss for $A_{50}$ equals −244!).

**Table 10:** Results obtained for different methods (b = 1, s = 5). *Source*: Prepared by the author

Examples$\backslash$methods	2-crit(H+B)	Hurwicz	SAPO $(\hbox {d}^{\mathrm{min}}=0.4$ $\hbox {d}^{\mathrm{max}}=0.4$)	SF+AS	
Ex IX ($\upalpha = 0.9$)	$\hbox {A}_{1}$ (1.0)	$\hbox {A}_{1}$ (1.0)	$\hbox {A}_{1}$ (1.0)	$\hbox {A}_{1}$ (1.0)	$\hbox {S}_{1}$
	$\hbox {A}_{2}$ ($-$2.5)	$\hbox {A}_{2}$ ($-$3.4)	$\hbox {A}_{2}$ ($-$3.4)	$\hbox {A}_{2}$ ($-$4.0)	
	$\hbox {A}_{3}$ ($-$6.5)	$\hbox {A}_{3}$ ($-$7.8)	$\hbox {A}_{3}$ ($-$7.8)	$\hbox {A}_{3}$ ($-$9.0)	
	$\hbox {A}_{4}$ ($-$11.0)	$\hbox {A}_{4}$ ($-$12.2)	$\hbox {A}_{4}$ ($-$12.2)	$\hbox {A}_{4}$ ($-$14.0)	
Ex X ($\upalpha = 0.5$)	$\hbox {A}_{1}$ (1.0 / 0.00)	$\hbox {A}_{1}$ (1.0)	$\hbox {A}_{1}$ (1.0)	$\hbox {A}_{2}$ (2.0)	$\hbox {S}_{2}$
	$\hbox {A}_{2}$ (0.5 / 3.00)	$\hbox {A}_{2}$ ($-$1.0)	$\hbox {A}_{2}$ ($-$0.5)	$\hbox {A}_{1}$ (1.0)	
	$\hbox {A}_{3}$ ($-$1.5)	$\hbox {A}_{3}$ ($-$3.0)	$\hbox {A}_{3}$ ($-$2.63)	$\hbox {A}_{3}$ ($-$3.0)	
	$\hbox {A}_{4}$ ($-$5.0)	$\hbox {A}_{4}$ ($-$5.0)	$\hbox {A}_{4}$ ($-$3.5)	$\hbox {A}_{4}$ ($-$8.0)	
Ex XI ($\upalpha = 0.62$)	$\hbox {A}_{1}$ (1.0)	$\hbox {A}_{1}$ (1.0)	$\hbox {A}_{1}$ (0.94)	$\hbox {A}_{1}$ (1.00)	$\hbox {S}_{2}$ , $\hbox {S}_{1}$
	$\hbox {A}_{2}$ ($-$0.11)	$\hbox {A}_{2}$ ($-$1.72)	$\hbox {A}_{2}$ ($-$1.34)	$\hbox {A}_{2}$ (0.98)	
	$\hbox {A}_{3}$ ($-$2.52)	$\hbox {A}_{3}$ ($-$4.44)	$\hbox {A}_{3}$ ($-$4.16)	$\hbox {A}_{3}$ ($-$4.02)	
	$\hbox {A}_{4}$ ($-$6.23)	$\hbox {A}_{4}$ ($-$7.16)	$\hbox {A}_{4}$ ($-$4.64)	$\hbox {A}_{4}$ ($-$9.02)	
Ex XII ($\upalpha = 0.1$)	$\hbox {A}_{2}$ (1.5 / 3.00)	$\hbox {A}_{4}$ (2.2)	$\hbox {A}_{4}$ (2.2)	$\hbox {A}_{4}$ (4.0)	$\hbox {S}_{4}$
	$\hbox {A}_{3}$ (1.5 / 5.74)	$\hbox {A}_{3}$ (1.8)	$\hbox {A}_{3}$ (1.8)	$\hbox {A}_{3}$ (3.0)	
	$\hbox {A}_{1}$ (1.0)	$\hbox {A}_{2}$ (1.4)	$\hbox {A}_{2}$ (1.4)	$\hbox {A}_{2}$ (2.0)	
	$\hbox {A}_{4}$ (1.0)	$\hbox {A}_{1}$ (1.0)	$\hbox {A}_{1}$ (1.0)	$\hbox {A}_{1}$ (1.0)

**Table 11:** Payoff matrix, sums of “dominance cases” and intervals for $\beta$ (for b = 1, s = 5). *Source*: Prepared by the author

Scenarios	Alternatives				Sum of “dominance cases” ($d_{i}$)	Interval for $\beta$
	$A_{1 }$	$A_{2 }$	$A_{3}$	$A_{4 }$		
$S_{1}$	1	$-$4	$-$9	$-$14	0	[0.00, 0.14]
$S_{2}$	1	2	$-$3	$-$8	3	]0.43, 0.57]
$S_{3}$	1	2	3	$-$2	5	]0.71, 0.86]
$S_{4}$	1	2	3	4	6	]0.86, 1.00]

In the author’s opinion each decision rule presented in Tables 8 and 10 may be applied to decision making under complete uncertainty. However, the choice of a suitable procedure should depend on many diverse factors:

1. Hurwicz rule may be certainly used when the distribution of payoffs connected with a given alternative is (almost) symmetric and decision variants have quite similar dispersions (measured e.g. by the standard deviation).
2. SAPO rule may be used when the distribution of payoffs connected with a given alternative is symmetric or asymmetric. The approach is addressed to DMs who focus on the specificity of the subsets of extreme values and who do not care about the frequency and the level of intermediate profits. In the case of SAPO, decision variants should have quite similar payoff dispersions.
3. 2crit(H+B) rule may be used when the distribution of payoffs connected with a given alternative is symmetric or asymmetric. The procedure is addressed to DMs who are willing to analyze the specificity of the whole payoff set (range, frequency, dispersion) and that is why, 2crit(H+B) can be even applied to decision variants having significantly different outcome dispersions.
4. SF+AS rule may be used when one has a strong belief in the predictive power of one’s declared coefficient of optimism (pessimism) since in this case the alternative is selected on the basis of one (sometimes more) concrete state of nature. Thus, there is a strong assumption that just that particular scenario will occur. And in the context of one-shot decisions such an assumption is totally justifiable, but in problems where particular decisions are characterized by considerably diverse payoff dispersions, we encourage to apply SF+AS quite cautiously.

In connection with the conclusions made above, we venture to state that since:

- in the NP problem alternatives differ essentially from others in terms of payoff dispersion,
- problems with innovative products are characterized by a high level of uncertainty (because there are no historical data allowable to estimate the payoff matrix, i.e. to estimate parameters $$c_{1}$$, $$c_{2}$$ and $$c_{3})$$,

the 2-criteria (H+B) rule may be the best procedure, especially if the DM intends to control all profits related to a given order quantity (not some of them). However, the final choice of a proper decision rule should depend on DM’s preferences. SF+AS is a better approach if the DM treats payoffs connected with an alternative as a sequence (i.e. he or she pays attention to the position of a given outcome in the profit matrix). On the other hand, Hurwicz, SAPO and 2crit(H+B) rules treat those payoffs as a set (the order is not crucial—it is not important to which state of nature particular profits are related).

At the end of that section, we would like to present decisions recommended by 2crit(H+B) and Hurwicz rules for data given in Table 3, second column and $\alpha \in [0,1]$. Results are gathered in Tables 12 and 13. The last two columns in these tables show the minimal and maximal available profit on condition that the suggested decision variant is selected.

**Table 12:** 2crit(H+B) values for data given in Table 3 (2nd column, $b=3$, $s=3$), $\alpha \in [0,1]$. *Source*: Prepared by the author

Alpha	Alternatives				Minimal profit	Maximal profit
	$A_{1}$	$A_{2}$	$A_{3}$	$A_{4}$		
1.00	**3**.**00**	0.00	$-$3.00	$-$6.00	3	3
0.95	**3**.**00**	0.82	$-$1.64	$-$4.36	3	3
0.90	**3**.**00**	1.50	$-$0.50	$-$3.00	3	3
0.85	**3**.**00**	2.08	0.46	$-$1.85	3	3
0.80	**3**.**00**	2.57	1.29	$-$0.86	3	3
0.75	**3**.**00**	**3**.**00**	2.00	0.00	3 or 0	3 or 6
0.70	(3.00)	**3**.**38**	2.63	0.75	(3 or) 0	(3 or) 6
0.65	3.00	**3**.**71**	3.18	1.41	0	6
0.60	3.00	**4**.**00**	3.67	2.00	0	6
0.55	3.00	**4**.**26**	4.11	2.53	0	6
0.50	3.00	**4**.**50**	**4**.**50**	3.00	0 or $-$3	6 or 9
0.45	3.00	(4.58)	**4**.**74**	3.47	(0 or) $-$3	(6 or) 9
0.40	3.00	4.67	**5**.**00**	4.00	$-$3	9
0.35	3.00	4.76	**5**.**29**	4.59	$-$3	9
0.30	3.00	4.88	**5**.**63**	5.25	$-$3	9
0.25	3.00	5.00	**6**.**00**	**6**.**00**	$-$3 or $-$6	9 or 12
0.20	3.00	5.14	(6.43)	**6**.**86**	($-$3 or) $-$6	(9 or) 12
0.15	3.00	5.31	6.92	**7**.**85**	$-$6	12
0.10	3.00	5.50	7.50	**9**.**00**	$-$6	12
0.05	3.00	5.73	8.18	**10**.**36**	$-$6	12
0.00	3.00	6.00	9.00	**12**.**00**	$-$6	12

Bold numbers represent the highest values of the 2crit(H$+$B) indices

**Table 13:** Hurwicz values for data given in Table 3 (2nd column, $b=3$, $s=3$). $\alpha \in [0,1]$. *Source*: Prepared by the author

Alpha	Alternatives				Maximal loss	Maximal profit
	$A_{1}$	$A_{2}$	$A_{3}$	$A_{4}$		
1.00	**3**.**00**	0.00	$-$3.00	$-$6.00	3	3
0.95	**3**.**00**	0.30	$-$2.40	$-$5.10	3	3
0.90	**3**.**00**	0.60	$-$1.80	$-$4.20	3	3
0.85	**3**.**00**	0.90	$-$1.20	$-$3.30	3	3
0.80	**3**.**00**	1.20	$-$0.60	$-$2.40	3	3
0.75	**3**.**00**	1.50	0.00	$-$1.50	3	3
0.70	**3**.**00**	1.80	0.60	$-$0.60	3	3
0.65	**3**.**00**	2.10	1.20	0.30	3	3
0.60	**3**.**00**	2.40	1.80	1.20	3	3
0.55	**3**.**00**	2.70	2.40	2.10	3	3
0.50	**3**.**00**	**3**.**00**	**3**.**00**	**3**.**00**	3, 0, $-$3 or $-$6	3, 6, 9 or 12
0.45	3.00	3.30	3.60	**3**.**90**	$-$6	12
0.40	3.00	3.60	4.20	**4**.**80**	$-$6	12
0.35	3.00	3.90	4.80	**5**.**70**	$-$6	12
0.30	3.00	4.20	5.40	**6**.**60**	$-$6	12
0.25	3.00	4.50	6.00	**7**.**50**	$-$6	12
0.20	3.00	4.80	6.60	**8**.**40**	$-$6	12
0.15	3.00	5.10	7.20	**9**.**30**	$-$6	12
0.10	3.00	5.40	7.80	**10**.**20**	$-$6	12
0.05	3.00	5.70	8.40	**11**.**10**	$-$6	12
0.00	3.00	6.00	9.00	**12**.**00**	$-$6	12

Bold numbers represent the highest values of the Hurwicz indices

We can notice that, depending on the coefficient of pessimism, each alternative ($A_{1}$, $A_{2}$, $A_{3}$ or $A_{4}$) may be recommended by the 2crit(H+B) procedure, while Hurwicz rule suggests only $A_{1}$ or $A_{4 }$ (apart from one case: ${\alpha }=0.50$). This is due to the fact that 2crit(H+B) indices depend on all values connected with a given decision and Hurwicz indices depend only on extreme values. It is worth underlining that suggestions provided by Hurwicz approach are quite astonishing, because according to that procedure:

- $A_{1}$ is the best one for both extreme pessimists (${\alpha }=1.00$) and moderate pessimists (${\alpha }=0.51$),
- $A_{4}$ is the best one for both extreme optimists (${\alpha }=0.00$) and moderate optimists (${\alpha }=0.49$),
- final recommendations are significantly sensitive to the coefficient of pessimism in the interval $\alpha \in [0.49,0.51]$.

In the case of 2crit(H+B) procedure, recommendations change more often, but those changes are less radical. The range of possible outcomes increases gradually (not violently) with the decrease of the coefficient of pessimism.

## Conclusions

We formulate a new problem. i.e. the non-repeated newsvendor problem under complete uncertainty (NPCU) for innovative and small life cycle products. The problem at hand is tackled for the first time in this work. We also make an attempt to find a suitable procedure enabling one to recommend an appropriate alternative. The 2-criteria (H+B) rule, proposed and described in the paper, may be a quite comfortable and comprehensive decision tool in the newsvendor problem under complete uncertainty for new and small life cycle products since it takes into account DM’s preferences and the very specific dispersion of payoffs (asymmetry, range, frequency of particular values). It does not require any information about the likelihood (which is justifiable in the case of innovative products) and it is rather simple to use. The 2-criteria (H+B) rule may be helpful in any uncertain decision problem (not merely NPCU), especially in the case of a considerable extreme payoffs differential.

In the paper, we analyzed cases for which $b-s=4$, $b-s=0$ and $b-s=-4$ (*b*—unit profit, *s*—unit loss). If we investigate problems where the absolute difference between the unit profit and the unit loss is much higher (e.g. 10, 50, 100 or 1000). we will just notice that the ranges of payoffs for particular alternatives increase and that the differences between standard deviations increase as well, which only confirms the necessity to use a procedure considering the payoff dispersion.

We would like to stress that in this paper we were trying to develop a proper method for NPCU. Nevertheless, the final choice of decision rules should be in the competence of the DM. Some decision makers may intend for instance to combine the hybrid of Hurwicz and Bayes rule (used in this research) with an other dispersion measure (instead of the standard deviation applied in the contribution). Other DMs may be for example interested in using the approach suggested by Perez et al. (2015).

In the future it would be desirable to analyze extended newsvendor models in the context of complete uncertainty, e.g. models with utility functions, models with different pricing policies or multi-item newsvendor models. A very interesting topic worth investigating concerns NP with interval payoffs for each combination scenario–alternative (i.e. demand–order). Note that there are also such NP models for which complete uncertainty does not occur in the whole decision making process. For instance, in the multi-period NP our knowledge about future demand increases with time. Hence, in subsequent periods we do have historical data and we are able to estimate the probability distribution. That means that in the multi-period NP (repeated NP) with innovative products the problem is solved under complete uncertainty in the first period and then it is solved under partial uncertainty or risk in the next periods.
